# A retrospective study of Crimean-Congo hemorrhagic fever in Iraq

**DOI:** 10.4314/ahs.v24i1.8

**Published:** 2024-03

**Authors:** Doaa Adnan Shaker, Muna Tawfeeq Abd, Nawar Jassim Alsalih, Sinan Ghazi Mahdi, Mohenned Alsaadawi, Ihab Raqeeb Aakef, Tareq Jafaar Aljandeel

**Affiliations:** 1 Communicable Diseases Control Center, Zoonotic Diseases section, Baghdad, Iraq; 2 College of Veterinary Medicine, AL-Muthanna University, Samawah, Iraq; 3 Alhadi College University

**Keywords:** Crimean–Congo hemorrhagic fever, Iraq, tick

## Abstract

**Background:**

CCHF is transmitted via ticks biting and directly by contact with tissue or blood of infected patients or viremic animals. This study intends to determine the occurrence of CCHF in Iraq between 2015 and 2019.

**Methods:**

This study was designed as a retrospective and descriptive cross-sectional study. It was approved the occurrence of CCHF in Iraq with relation to some epidemiological and demographic data reported in the Iraqi Communicable Diseases Control Center (CDC)/zoonotic diseases section between 2015-2019.

**Results:**

Out of 206 suspected cases, only 17 were diagnosed as CCHF with a total fatality ratio of 52%, 25%, and 80% in 2015 and 2018 respectively. However, no mortality was reported during 2016, 2017, and 2019. The mean age of the patients was 33 years± 18 SD, in males mainly (76%). Moreover, the risk groups were 29 %, 23 %, 18 % 12 %, and 6 % for butchers, animal dealers, gainers, both housewives and students and children respectively.

**Conclusion:**

Strict precautions and precise surveillance should be implemented to control the disease and protect the community by improving the diagnosis and treatment of CCHF. The authors recommend another future study to detect the genotyping and sequencing of CCHFV in Iraq.

## Introduction

Recently, the need of doing prevalence studies of microbial pathogens increases in Iraq as it can focus on the medical important diseases either in humans or animals 1–6. CCHF is a viral zoonotic disease recorded in many countries in Asia, Africa, the Middle East, and Europe. The infected animals were asymptomatic; however, it develops a severe disease in humans 7. Crimean-Congo hemorrhagic fever orthonairovirus (CCHFV) is an arbovirus; a member of the genus Orthonairovirus (Nairoviridae family). CCHF was first recognized in the Crimean Peninsula during the mid-1940s among agricultural workers 8. The bite of infected ticks or direct contact with blood or tissues of infected patients or animals is considered the transmission ways of CCHFV to humans 9, 10, causing a serious illness with fatality up to 30% 10. It is known that any deformation in blood cells can cause highly serious disturbance in their functions 11. At least 31 different tick species can transmit CCHFV 12. Hyalomma species' wide distribution which includes different types of environments such as dessert can relate to their capacity to adapt and manage their survival in thus environments 13. It can work as a vector for different diseases such as CCF 14. Hyalomma spp. has been documented as the most virus vector 10. Symptoms of CCHF include fever, fatigue, headache, loss of appetite, myalgia, abdominal pain, hemorrhage, and severe thrombocytopenia 15,9. Healthcare professionals are at significant risk of infection, specifically during the treatment of patients with hemorrhages from various body sites 9.

In Iraq, in 1979, CCHF was documented for the first time 16. In 2010, the disease re-emerged in eight Iraqi provinces. The transmission of CCHF occurred because of contact with the blood and tissues of infected animals 17. Contact with slaughtered sheep at home was recorded as one of the transmission methods in one patient only 17. The rapid increase in CCHF cases in 2010 has been noticed, therefore the Iraqi CDC initiated a number of awareness programs conducted by both the public and the medical community incorporating the Ministry of Agriculture (the responsible institution for animal welfare in Iraq) in order to control ticks by dipping and spraying animals 18 or inventing new therapeutic agents such as using herbal plants which can reduce the amount of environmental pollution 19. In addition, there are an increased need to develop the knowledge about the role of immune system against this which needs to use ELISA or histopathology that should clear description for the pathogenic diseases 20–22.

No more papers regarding CCHF in Iraq were published. Therefore, the authors designed this descriptive cross-sectional retrospective study to study the occurrence of CCHF in Iraq in relation to some epidemiological and demographic data such as the age, gender, occupation, season, and provinces reported in Iraq CDC/ zoonotic diseases section from 2015-2019. Additionally, recommendations and management procedures can be used as a reference source to enhance the control measurements.

## Methods

### Study design

A retrospective study and a descriptive cross-sectional study were performed by using some demographic and epidemiological data, that were collected from Iraq CDC/zoonotic section. Case investigation forms were received from CDC branches in other Iraqi provinces by using Microsoft Excel till 2018. After that, the documented system in the zoonosis section has been changed, and Epi info7 software was adopted. The suspected and confirmed cases reported from January 2015 to December 2019, were included in this study. Standard Case Definition Form was used for suspected cases which included: sudden onset of illness, fever, and history to exposure as contact with livestock, tick bites, or contact with confirmed cases, through the previous 14 days before the onset of symptoms or severe clinical disease with progression to hypotension or shock, and the confirmed cases define as suspect cases with positive for laboratory test ELISA or PCR.

A total of 206 suspected cases were recorded during the last five years. The disease was confirmed by using ELISA to detect IgM or IgG antibodies in serum or Real-time polymerase chain reaction (RT-PCR) for viral nucleic acid detection. The confirmed tests were done in the Central Public Health Laboratory (CPHL), the reference lab for Hemorrhagic Fever in Iraq. The seventeenth confirmed cases of CCHF included in the study occurred in different locations in Iraq.

### Real-time PCR technology

The RealStar® CCHFV RT-PCR Kit 1.0 (Altona Diagnostics, Germany) was used to detect Crimean-Congo hemorrhagic fever (CCHF) virus-specific RNA has been used.

### RT-PCR protocol

140 µl of serum was used to extract total RNA using the QIAamp® Viral RNA Mini Kit (QIAGEN, Germany). Then the steps instructed in the extraction kit protocol were followed.

### Statistical analysis and mapping

Data were analyzed by using Microsoft Excel 10 and draw charts, and GraphPad Prism 9. The statistical analysis was done by using Qi square, P≤0.05. This study used QGIS 3.10.6 software and then baseline data was created using the QGIS Base map Open Street Map service.

## Results

**Figure 1:**
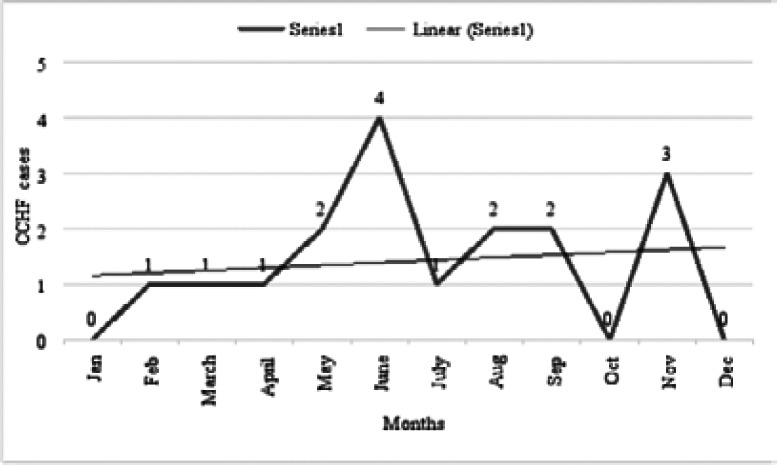
Trend and monthly distribution of CCHF cases in Iraq from 2015-2019

**Figure 2:**
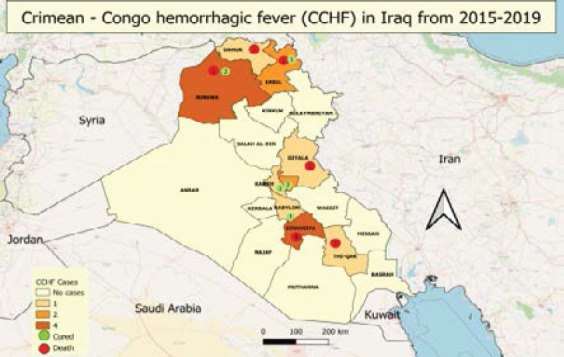
Geographic distribution of CCHF in Iraq from 2015-2019

**Figure 3:**
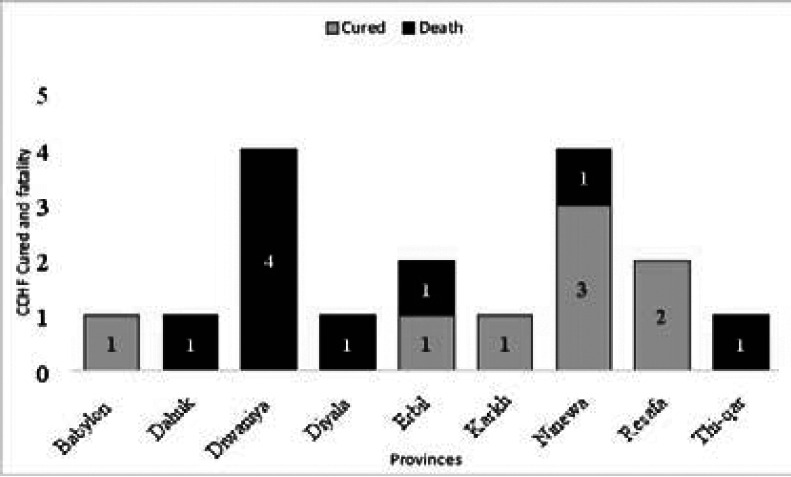
Cured and fatality of CCHF in Iraqi provinces from 2015-2019

Iraqi CDC monitors CCHF cases, depending on immediate notification by using a case-based investigation questionnaire. From 2015 to 2019, the CDC system received 206 suspected cases, all of these cases were tested by using ELISA or RT-PCR. Only seventeen cases were confirmed as positive with a total infection rate of 8.2%. Four cases in 2015, ten cases in 2018, and three cases in 2019. These cases are reported in different seasons (Figure 1). Geographically, suspected cases have been reported from all provinces, however, the positive cases were recorded only in half (n=9) provinces during the last five years (Figure 2), with the greatest proportions of cases reported from Diwaniya and Ninewa, (Figure 3).

**Figure 4:**
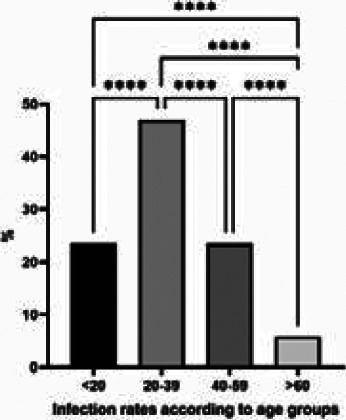
Distribution of Crimean-Congo hemorrhagic fever confirmed cases according to age groups. The infected cases appeared to be differently distributed according to age groups of infected individuals. The differences were analysis using Qi square, P≤0.05

**Figure 5:**
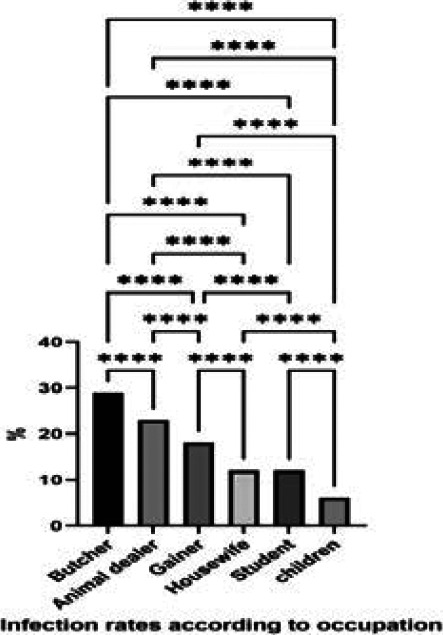
Distribution of Crimean-Congo hemorrhagic fever confirmed cases according to patients' occupation in Iraq 2015-2019. The infected cases appeared to be differently distributed according to occupation of infected individuals. The differences were analysis using Qi square, P≤0.05

**Figure 6:**
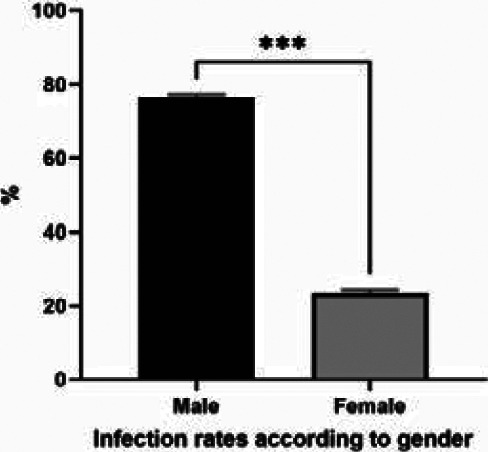
Distribution of Crimean-Congo hemorrhagic fever confirmed cases according to the gender. The infected cases appeared to be differently distributed according to gender of infected individuals. The differences were analysis using Qi square, P≤0.05

The reported cases ages ranged from 4 to 68 years. About half of the cases (47%) were reported among ages (20-39) years old, (23%) of cases among less than 20 years and (40–59) years old individuals, and (6%) in patients who are more than 60 years old (Figure 4). For the occupation, most of those reported were butcher and animal dealer, (29%) (23%) respectively, gainer (18%), both housewives and students (12%), and children (6%) (Figure 5). The infection rates showed significant differences between males and females (76% and 24 respectively) (Figure 6). The incidence rate of infection with CCHF in Iraq in 2015, 2018, and 2019 was 0.01, 0.026, and 0.007, respectively. Among confirmed cases, the total fatality ratio (CFR) was (52%) (25% in 2015, 80% in 2018), and no mortality in 2016, 2017, and 2019. The total CFR among suspected cases was 7% (11% in 2015, 4% in 2018, and 22% in 2019 during the same period) while in 2016, and 2017, there are no death occurred.

The symptoms of confirmed cases were fever and hemorrhage after few days from fever onset, petechial, gingival, and site of injection hemorrhage. Supportive treatment therapy can include intravenous fluids, analgesia, platelet and/or blood transfusions provided for most cases, and antiviral therapy (Ribavarin).

## Discussion

CCHFV is recognized as one of the major emerging zoonotic diseases which can cause high case fatality rates and can be transmitted from human to human. There are many factors affecting the emergence of CCHF including climate change, ecological changes, and land cover types, rises in the population and survival of tick and virus circulation 23, increased populations of wild animals, domestic and transnational animal movements, and the carrying of the virus by migratory birds that infested with ticks carrying the virus 24. These factors have complicated interactions which affect both the onset and the prevalence of the virus 25. There are many limitations in the surveillance, diagnosis, and detection of the disease. However, serological and molecular epidemiological studies in the endemic area can be valuable to evaluate the risk of CCHF in humans. A serological survey in Iran from 1975 to 1999, confirmed that 25–80% of the sheep were CCHFV seropositive 26.

In the World Health Organization Eastern Mediterranean Region (WHO EMR) countries, including Iraq, the incidence of CCHF is rapidly growing 27. Human cases of CCHF are reported annually to CDC\Iraq where (17) cases were confirmed as CCHF from 2015-2019 out of (206) suspected cases (the infection rate is 8.2%). The cases may be much more than this number in theb fact. This can relate to insufficient diagnostic laboratory kits for CCHF diagnosis, particularly in rural places. In addition, as it is known that the health system in Iraq is exhausted, the process of recording and reporting cases was not at the required level, since many areas were suffering from fighting wars with terrorist organizations that started in 2014 whose effects continued until 2020. Therefore, the process of reporting cases or their laboratory diagnosis was very weak, since the disturbances were mainly in rural areas, where infection rates are expected to be high due to contact with risk vectors. CFR was (52%) among confirmed cases which is higher than the fatality rate of reported cases in 2010 in Iraq 18, and the fatality rate documented in other countries such as Pakistan Turkey, Iran, Afghanistan, and Oman (Umair *et al.* 2020; Gunes et al. 2009; Keshtkar-Jahromi et al. 2013; Niazi and Mohammad Jawed Jawad, Ahmad Amirnajad, Peter A. Durr 2019) 15. The high fatality rate may relate to the health system quality in the diagnosis and treatment of infected individuals and the comorbidities that affect patients accompanied by the viral load. The mean of CCHF human fatality rates differs from one region to others affected by the sensitivity of people to CCHFV infection or reduced pathogenicity and the distribution of CCHFV clades. In Africa, the CCHF infection rate was (22.0 %) which is lower than in Asia (33.5 %) and Europe (33.8 %) [32], while the fatality rate in suspected cases was (7%). The peak of cases reported in June and November is different from the findings of seasonal occurrence of CCHF in 2010 where the majority of cases occurred from the end of April to early July 18. Nevertheless, other studies usually reported CCHF cases during the summer 33, 31, 28.

The study showed there are significant differences between males and females which is similar to many studies 33, 18, 34, 31. This could be attributed to the nature of social life in Iraq, which depends mainly on men in performing basic life actions such as doing business outside the home, especially those that require high physical efforts such as farming or animal husbandry. Therefore, men are more susceptible to disease because of their frequent contact with risk factors that can transmit the disease. While women tend to work indoors or be a part of governmental and office jobs, which require less effort and do not need to deal with disease-carrying factors such as dealing with animals.

The most prevalent human activities implicated in CCHF were agricultural, animal husbandry, health care and abattoir workers, farmers, and housewives. Based on the occupation of infected people results showed that butcher's morbidity was (29%); this rate was much higher than in other studies 29 (Niazi *et al.*, 2019) 35, irregular unrestricted slaughtering without using suitable individual protective equipment in addition to tick bites 36, and living near the livestock 23 considered important risk factors to get CCHF infection.

The study revealed that (47%) were reported among (20-39) years old. This age group represents the working age group in society 37; therefore, it is normal to see the highest infection rate in this age group.

Other studies reported that the geographic distribution of CCHF cases was the same as recorded in our study 17, 18. In Diwaniya and Ninewa, the highest number of infected cases were reported. Diwaniya has huge agricultural areas which encourage breeding the animals in large numbers and flocks. This can interfere with the high percentage of CCHF infection rates in this city. Ninewa, in the north of Iraq, has long border areas with Turkey. The prevalence rate of CCHF in Turkey was 12.8% 29. In addition, another Turkish study suggested that the borders with Iraq are uncontrolled which allow for entry and exit of the flocks to Ninewa which can increase the occurrence of CCH-FV due to the unregulated animal movements in these areas that may have played a role in the spread within the country and to neighbouring countries 25. Moreover, the import of infected livestock illegally from neighbouring countries was a cause of an outbreak of CCHF in Iran 38. In conclusion, the uncontrolled animal movement between the Iraqi areas and neighbouring countries may be considered one of the causes of rising the incidence of CCHF in Iraq.

Frequent symptoms of documented confirmed cases were fever and hemorrhage after one day of fever onset, petechial, gingival, and site of injection hemorrhage with thrombocytopenia. Symptoms were similar to the results of another study 17. Another study in other countries reported fever, malaise, headache, abdominal pain, myalgia, nausea, vomiting, thrombocytopenia, and bleeding manifestations were the most common signs 15, 21, 34, 39. The clinical type of infection can be affected by factors such as host immune response, viral load, and lack of certain receptors 32. 100% of the patients infected with CCHF suffer from thrombocytopenia, 80% of them had leukopenia, and only 30% of infected individuals had anemia 9.

It is clear from the obtained results that RT–PCR can be used as a good technique to establish a trusted diagnostic technique during the first two weeks of illness. In instances where negative RT–PCR results are obtained during the acute stage of the illness; it would be quicker and theoretically more accurate to attempt second round PCR with nested primers rather than Southern blots. However, it is necessary to use RT–PCR in combination with other tests to get higher accuracy. In addition, protein recombination techniques 40 can be used in preparing high-efficacy vaccines and diagnostic kits for local strains of CCHF. It is recommended to do more immunological studies to explain how the virus can evade the immune response as it is referred to the role of complement in the pathogen's infections 41, 42.

## Conclusion

Iraq can be considered an endemic area for CCHF. Therefore, it is preferable to implement control programs in cooperation with the veterinary side to control the disease in animals and protect the community, increase health promotion between risk groups, especially those who work in contact with animals, and diagnose other types of hemorrhagic fever that spread in neighbouring countries and the region to confirm suspected cases. Advanced studies are needed to detect the genotyping and sequencing of CCHFV in Iraq.
